# Editorial: Effects of radiation therapies on brain metastases

**DOI:** 10.3389/fonc.2023.1196143

**Published:** 2023-04-13

**Authors:** Rupesh Kotecha, John H. Suh, Minesh P. Mehta

**Affiliations:** ^1^ Department of Radiation Oncology, Miami Cancer Institute, Baptist Health South Florida, Miami, FL, United States; ^2^ Herbert Wertheim College of Medicine, Florida International University, Miami, FL, United States; ^3^ Deparment of Radiation Oncology, Taussig Cancer Institute, Cleveland Clinic, Cleveland, OH, United States; ^4^ Cleveland Clinic Lerner College of Medicine of Case Western Reserve University, Cleveland, OH, United States

**Keywords:** brain metastases, radiation therapy, neurocognition, stereotactic radiosurgery, whole-brain radiotherapy

Brain metastasis represents the most common malignancy diagnosed in adults with approximately 15 to 30% of patients with solid tumors at risk of developing intracranial dissemination at some point during their disease course (1). In the multi-disciplinary management of patients with brain metastasis, radiotherapy is often a substantial component of either definitive or adjunctive management. In addition to the development of novel radiotherapy advances, the neurocognitive function has been the subject of contemporary research, with an emphasis on risk mitigation and amelioration of toxicity severity (Lehrer et al.). There have been several recent advances in our understanding of the role of radiotherapy in the management of brain metastasis, including a re-evaluation of the biology of brain metastasis and its impact on treatment decisions, effect on neurocognitive function, and long-term outcome comparisons between stereotactic radiosurgery (SRS) and whole-brain radiotherapy (WBRT), which is the theme of this Research Topic collection.

Khan et al. provide a comprehensive overview of our current understanding of the role of SRS and WBRT in patients with brain metastasis by reviewing the literature specifically for the two most common tumors associated with brain metastasis, lung and breast cancer, as well as radioresistant tumors (i.e. renal cell carcinoma and melanoma); they also focus on the unique scenario of a single brain metastasis. For patients with non-small cell lung cancer (NSCLC), they recommend SRS alone for the majority of patients but also consider WBRT as an upfront diagnosis for selected patients with better-expected prognosis given the results of updated subset analyses from the RTOG 9508 (2) and JROSG 99-1 trials (3). This was also the subject of one of the original research reports in this topic collection by Ni et al., who demonstrated improved survival, especially in those with limited intracranial disease (one to three lesions) or those receiving targeted therapy, in a large series of 684 NSCLC patients treated with or without the combinatorial approach. It is important to note, however, that a subset analysis from N0574 did not recapitulate this benefit (4) and the recent ASTRO (5) and ASCO (6) brain metastasis guidelines do not categorically recommend upfront combined modality treatment, which increases the risk for neurocognitive decline. The identification of patients with central nervous system (CNS) dominant disease, an example of such an exercise evaluated by Trikhirhisthit et al., is clearly needed to better select patients to categorically test the survival benefit hypothesis of combinatorial approaches, especially in the modern era where NSCLC patients receive intracranially active therapies such as immune checkpoint inhibitors and mutation-targeted drugs. In addition, for NSCLC patients with actionable targetable mutations, the lack of level-one comparisons of targeted agents with/without upfront radiotherapy has led to significant variations in clinical practice and deserves specific attention in future trials.

For patients with breast cancer brain metastasis, SRS is commonly used as an upfront treatment given the expected prognosis for this population; recently, combination approaches with molecularly targeted agents, such as Lapatinib and radiotherapy, have been performed (7). As newer systemic therapies become available, categorical data supporting the best possible approaches for integrating local and systemic approaches have become less data-driven and, hence, highly variable. A concept-illustrating example by Ying et al. describes an excellent clinical response with the combination of apatinib (a potent antiangiogenic agent directed at the vascular endothelial growth factor receptor-2) and WBRT. For radioresistant tumors such as renal cell carcinoma and melanoma, SRS is typically recommended given the poor disease control rates with WBRT, and as noted by Khan et al., these also represent a disease site where novel agents such as tyrosine kinase inhibitors with intracranial penetration and immune checkpoint inhibitors are actively being used. Finally, the authors note that although a rare circumstance in the modern era, the optimal approach for a patient with a single brain metastasis currently remains controversial. An “entire compartmental” approach has been recommended, with local therapy such as surgery or SRS, with WBRT. Although not commonly used today, this comprehensive approach was associated with long-term survival in our experience as well (8).

Another topic of interest is the role of SRS or WBRT following the resection of brain metastasis. Currently, national patterns of care data indicate that the majority of patients receive post-operative SRS (9). El Shafie et al. report their long-term outcomes in 101 patients treated with either SRS (n=50) or WBRT (n=51) at a single institution. Not surprisingly, larger cavity volume and incomplete resection were associated with an increased risk of local failure. Yet, overall, local control and survival were longer in patients treated with SRS. This is likely due to the retrospective nature of the study with inherent selection biases with regard to upfront treatment. It is important to note that the long-term outcomes from the N107c randomized trial revealed superior intracranial control with WBRT but less neurocognitive deterioration with SRS (10).

Transitioning to the topic of management of specific brain metastasis categories, the optimal SRS approach for patients with large brain metastasis remains in flux as single fraction SRS local control rates are suboptimal (11). To this end, Putz et al. report on a comparison of lesions larger than 5 mm treated with SRS (18 Gy 1 fraction) or fractionated stereotactic radiotherapy (FSRT, 40 Gy in 10 fractions) in a cohort of 120 patients treated to 190 brain metastasis. A longer time for local progression (22.9 months vs. 14.5 months) and a lower 12-month rate of radiation necrosis (3.4 vs 14.8%) were observed in favor of FSRT. Although the results with this unusual fractionation schedule were favorable compared to single fraction SRS, most clinical practices use fractionated schedules delivered in three to five fractions. Another approach, especially for large lesions, is to perform staged SRS where single fraction SRS is delivered with a 3- to 4-week interval between sessions to allow for tumor shrinkage. In a dosimetric study of staged SRS cases replanned with fractionated SRS (FSRS) equivalent doses, Cui et al. demonstrated that staged SRS allowed for a higher dose to be delivered to the tumor with a comparable dose to the surrounding brain parenchyma. Therefore, prospective comparisons between fractionated approaches, hypofractionated schedules, or staged treatments are clearly needed.

Ultimately, future randomized controlled trials are clearly needed to investigate the optimal combinatorial approach in an era of systemic therapies with CNS penetration and may challenge the long-standing dogma of how SRS is currently being utilized. Given the WBRT data in specific subsets of patients, hippocampal-avoidant WBRT may also remain part of this combinatorial approach.

## Author contributions

All authors listed have made a substantial, direct, and intellectual contribution to the work and approved it for publication.
